# Electrical properties of PVA/PANI/LiI composite membranes

**DOI:** 10.1038/s41598-025-30722-x

**Published:** 2025-12-18

**Authors:** A. A. Eissa, E. M. Kamar, M. A. Mousa, M. Sameeh

**Affiliations:** Chemistry Department, Faculty of Science, Benha University, Benha, 13511 Egypt

**Keywords:** Ionic conductivity, Electric modulus, PVA, Polymer electrolytes, Transference number, Chemistry, Energy science and technology, Materials science

## Abstract

This study centers on polyvinyl alcohol and polyaniline films infused with lithium iodide as polymer electrolyte membranes that conduct ions for various electrochemical applications. Electrolyte membranes made from a 90:10 weight percent blend of polyvinyl alcohol (PVA) and polyaniline (PANI) were synthesized with varying amounts of LiI (from 5 to 25% by weight) via solution casting. The samples were analyzed through X-ray diffraction (XRD), Fourier transform infrared (FT-IR), mechanical properties testing, AC impedance spectroscopy, and linear sweep voltammetry (LSV). X-ray diffraction results show that the amorphous nature of PVA enhances with more additives, and LiI incorporation leads to improved mechanical properties in composite samples. FTIR analysis highlighted interactions between PVA and electrolyte components. Notably, the composites’ elongation at break surpassed that of the undoped PVA film, reaching 108 ± 7.1% with increased LiI content. The membrane containing 20 wt% LiI salt showed the highest ionic conductivity of 4.45 × 10^–3^ S cm^−1^. This top-performing membrane displayed an ionic transference number of 0.991 and demonstrated electrochemical stability at 2.21 V. All composite membranes displayed peaks in the dielectric loss spectra. The dielectric properties of the composites indicate a significant connection between the fluctuations in conductivity and the changes in the mobility and concentration of charge carriers. The composite materials exhibited non-Debye characteristics regarding their dielectric properties.

**Supplementary Information:**

The online version contains supplementary material available at 10.1038/s41598-025-30722-x.

## Introduction

With the global population increasing and industrial activities expanding, the energy demand continues to rise^1^. This growing demand necessitates the development of more efficient, reliable, and sustainable energy storage solutions^2^. Electrolytes are considered key components in energy systems, such as supercapacitors, batteries, and fuel cells. There are two types of electrolytes: liquid and solid. Liquid electrolytes pose possible safety risks, including leakage, combustion, and corrosion effects. On the other hand, solid-state electrolytes provide advantages including negligible leakage, reduced flammability, stability under varying temperatures, ease of processing, minimal self-discharge, increased potential for power density, and enhanced cycling performance^3,4^.

Polymer electrolyte, as one of the solid electrolytes, refers to any macromolecular polymer chain with added ionic salt characterized by a significant ionic conductivity approximately of the order of 10^−6^ S cm^−1^^5^. They have advantages such as a natural seal, resistance to shock and vibration, and resistance to temperature and pressure variation. The main drawbacks of this type of electrolyte are (i) low ambient conductivity and (ii) high interfacial resistance with electrodes. The above drawbacks are to be rectified by different approaches such as blending of two polymers^6,7^, plasticizing the polymer matrix using liquid additives^8,9^, incorporation of nano-sized fillers into polymer matrix^10–13^, etc.

Poly (vinyl alcohol) (PVA) is one of the synthetic polymers that has attracted considerable interest in the development of polymer electrolyte membranes. It is an environmentally friendly and reasonably priced polymer with strong film-forming properties, high hydrophilicity, and chemical resistance^14,15^. The oxygen in the PVA makes it polar, which promotes the formation of films with a neatly arranged, tightly spaced network structure of hydrogen bonds^16^. However, the main drawback to using PVA membranes in energy storage devices, as mentioned above, is their inferior conductivity. Various strategies can be employed to circumvent this**.** For instance, numerous hydrophilic OH functional groups allow the PVA polymer chain to undergo various chemical modifications, including substitution, grafting, crosslinking, and hydrogen bonding, especially when suitable dopant components are incorporated^17,18^. Consequently, studies have demonstrated that merging PVA with acidic polymers, such as polyaniline, is an effective approach for producing polyelectrolyte membranes because these membranes contain many ionizable groups^17–19^. It was also reported that many dopant salts can be used to synthesize PVA-based electrolytes^20^. Selecting the appropriate dopant salt during the preparation of the polymer electrolyte is crucial. The salts with significant lattice energy are difficult to break apart, whereas those with minimal lattice energy frequently undergo ion associations. In both scenarios, the ionic conductivity of the polymer electrolyte decreases because of a lower availability of free ions^21^.

The lattice energy of lithium iodide, 734 kJ/mol, lies within the suitable range required for making polymer electrolytes. Therefore, this study opted to utilize LiI as the main salt in PVA to create polymer electrolyte membranes. In addition, incorporating PANI as a plasticizer with PVA diminishes its crystalline characteristics, enhancing the mobility of polymer segments. This also leads to improved ion dissociation, which permits a greater number of charge carriers for ion transport within the electrolyte^22–30^.

In light of the information provided, this study aimed to create polymeric electrolyte membranes by blending polyvinyl alcohol (PVA) with PANI and varying amounts of lithium iodide (LiI). The membranes were produced using a solution-casting method. FT-IR and XRD techniques were utilized to investigate the overall structure of the membranes. At the same time, electrochemical assessments were conducted to analyze the electrical properties, including EIS, LSV, and Wagner’s polarization. This investigation also seeks to manage the ionic conductivity of the created polymer electrolytes and enhance their ionic conductivity by incorporating suitable fillers into the PVA.

## Materials and methods

### Materials

Pure PVA, with a molecular weight of about 75,000 g mol^−1^ and a hydrolysis degree of 87%, was sourced from Aladdin Reagent Company based in Shanghai, China. Sigma Aldrich supplied LiI (99.9%), acetone, HCl, and ammonium persulfate (APS), while N, N-dimethyl formamide (DMF, 99%) was acquired from Merck. Aniline (from SD Fine Chem., India) was vacuum-distilled prior to use. All the other chemicals were utilized in their original forms as received. All experiments were conducted using bidistilled water.

### Preparation of polyaniline

**Fig. 1:**
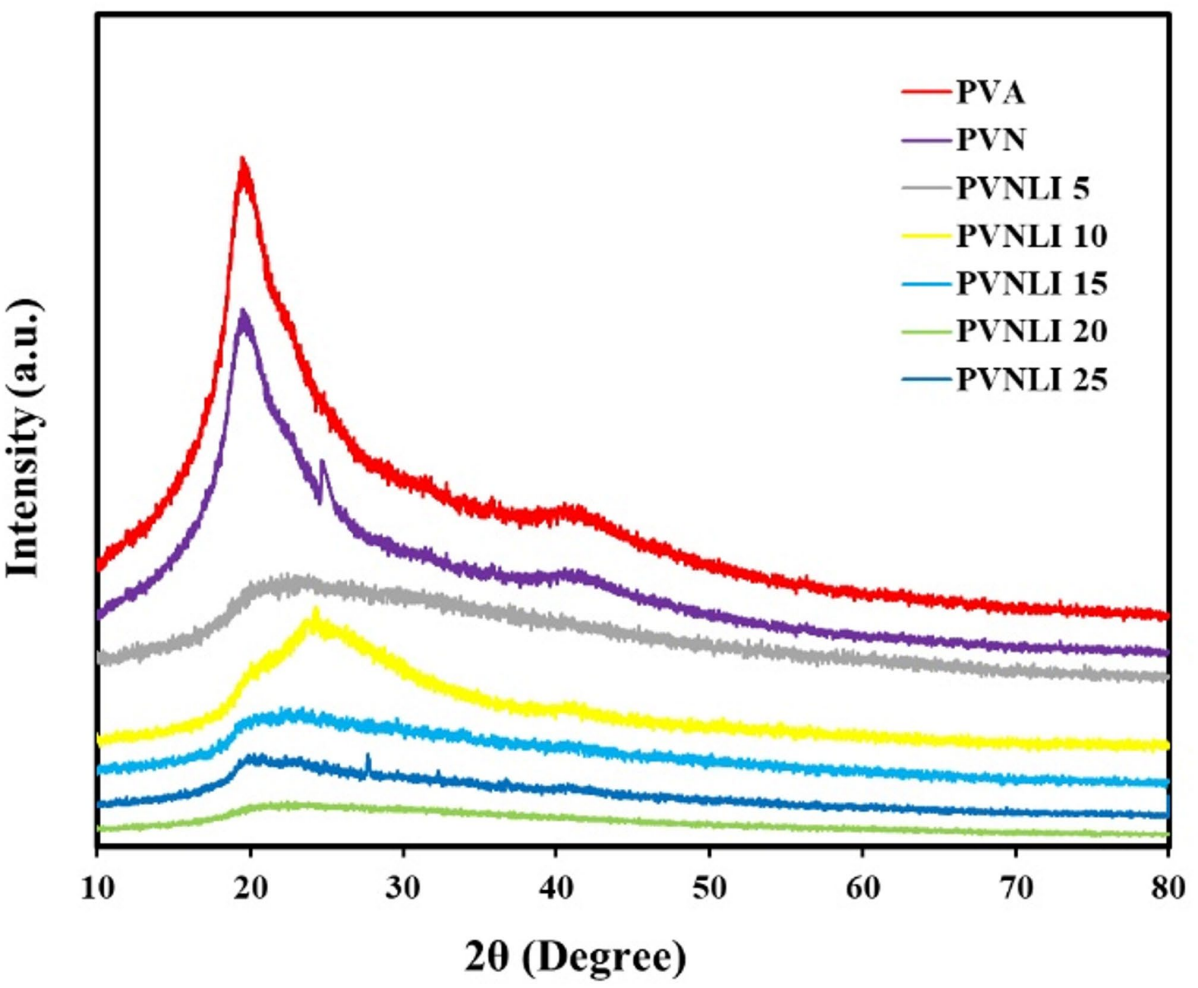
XRD of the investigated membranes.

Aniline hydrochloride was first produced by mixing 75 g of aniline with 80 ml of concentrated hydrochloric acid in an evaporating dish, followed by evaporating the mixture until dry. The resulting aniline hydrochloride was subsequently dried in an oven at a temperature range of 110–120 °C. Aniline hydrochloride (2.59 g, 20 mmol) was then dissolved in bidistilled water to provide 50 mL of solution within a volumetric flask. Ammonium peroxydisulfate (5.71 g, 25 mmol) was dissolved in 50 mL bidistilled water. Both solutions were permitted to remain at room temperature for one hour, combined in a beaker, stirred briefly, and then left undisturbed to allow for polymerization. The following day, the polyaniline (PANI) precipitate was gathered on a filter, rinsed with three 100 mL portions of 0.2 M HCl, and then washed with acetone. The polyaniline hydrochloride powder was allowed to dry in the air before being vacuum-dried at 60 °C. Figure 1S illustrate the depiction images of the synthesics methods.

### Preparation of electrolyte membrane

PVA powder (10% by weight) was placed in 30 mL of bidistilled water at 363 K for 2 h. A film with the appropriate thickness was subsequently formed by depositing the resulting solution onto a Petri dish. At room temperature, the solvent vaporized until a thin layer developed. A free-standing membrane was created when the resultant film was removed from the Petri dish. To fabricate the PVA-PANI membrane, the proportions of PVA and PANI were established as 90% and 10% (by weight) of the total composition, respectively. In a separate step, 10 mL of DMF solvent was blended with the designated quantities of PVA and PANI. The PANI was mixed in DMF for ten hours at room temperature. In the meantime, the PVA was solubilized by mixing it in DMF for four hours at a temperature of 70 °C. Following this, the PVA and PANI solutions were combined and stirred for two hours at 70 °C. The mixture was then allowed to cool before being stirred for another two hours to form the PVA-PANI base. The produced solution was placed onto a petri dish and air-dried. To produce a PVA-PANI-LiI membrane, specific amounts of LiI salt were incorporated into the PVA-PANI mixture, as indicated in Table 1. The process utilized to create the PVA-PANI matrix was also employed to synthesize PVA-PANI-LiI membranes. The LiI was incorporated into the PVA-PANI blend and stirred for two hours. The resulting blend was poured into a Petri dish with a diameter of 5 cm. The Petri dish was subsequently covered with filter paper and allowed to air dry at room temperature for 72 h. All the membranes formed a homogeneous thin film. The thickness of membranes was measured using a micrometer and found to be 150–200 μm for mechanical testing and 2–40 μm for electrical measurements.

**Table 1:** Code of the investigated membranes.

Sample Code	PVA (wt%)	PANI (wt%)	LiI (wt%)
PVA	100.0	–	–
PVN	90.0	10.0	–
PVNLi 5	85.5	9.5	5
PVNLi 10	81.0	9.0	10
PVNLi 15	76.5	8.5	15
PVNLi 20	72.0	8.0	20
PVNLi 25	67.5	7.5	25

### Characterization

X-ray diffraction analysis was performed using an X’Pert diffractometer employing Cu-Kα radiation (λ = 1.5405 Å) over a 2θ range of 10 – 80° with a step size of 0.02° and a scanning speed of 1°/min.

Fourier transform infrared spectroscopy (FTIR) measurements were conducted using a Bruker VECTOR 22 spectrometer at room temperature with a wavenumber resolution of 1 cm^−1^ using KBr pellets in the frequency range of 4000–400 cm^−1^.

Using the Instron 3300 mechanical testing apparatus, we assessed the mechanical characteristics of electrolyte films shaped in a rectangular format measuring 1 × 10 cm. Testing was conducted on three identical samples, adhering to ASTM D638, with gauge thickness between 0.10 and 0.20 mm. A steady crosshead speed of 2 mm per minute was utilized to get a consistent strain rate, allowing for the calculation of tensile strength, elongation at break, and elastik modulus^31,32^. Differences among the sample groups were analyzed using one-way ANOVA, a statistical method that verifies that the indicated trends are not merely the results of random fluctuation.

Complex impedance measurements were performed using an electrochemical analyzer (model: CHI608D, CH Instruments, Austin, USA). A thin membrane layer is placed between two specially designed stainless-steel electrodes. A spring was attached to apply a slight pressure between the electrodes to ensure good contact. Using impedance data acquired at a voltage of 5 mV, the real part (ε') and imaginary part (ε'') of the dielectric constant of the examined sample, as well as its AC-conductivity, were determined within the frequency range of 10^2^ – 3 × 10^5^ Hz. The transfer numbers of ions (t_ion_) and electrons (t_e_) at room temperature were assessed employing Wagner’s DC polarization method^33^, which involved monitoring the current over time for a polarized SS|electrolyte|SS cell linked to a digital multimeter (600 V, Model: CROWN B3 CT44051) and a stabilized potential set at 0.2 V using a digital Yescom DC Power Supply. The DY2300 potentiostate was employed to assess the membranes’ electrochemical stability window (ESW) by applying a voltage range of 0 to 3 V and a scan rate of 10 mV/s using the linear sweep voltammetry (LSV) technique.

## Calculation methods

The dc conductivity σ_dc_ values were computed using the following Eq.^34,35^:

1 $$\sigma_{{{\text{dc}}}} = {\text{ L}}/{\text{AR}}_{{\text{b}}}$$

where A represents the cross-sectional area of the membrane, σ indicates its conductivity, L signifies its thickness, and the bulk resistance (R_b_ ) is gotten from the low intersection of the real axis (Z') at a zero frequency.

The complex permittivity (ε^*^) of polymeric materials may be expressed as the real (ε') and imaginary components (ε'')^36^, where

2 $$\varepsilon^{*} = \, \varepsilon^{\prime } - {\text{ i}}\varepsilon^{\prime \prime }$$

The real dielectric constant (*ε*′) was calculated using Eq. (3).

3 $$\varepsilon^{\prime } = \left( {{\text{C}}/\varepsilon_{{\text{o}}} } \right){\text{x }}\left( {{\text{L}}/{\text{A}}} \right)$$

*C* denotes the capacitance of the sample, and *ε*_o_ = 8.85 × 10^−14^ Fcm^−1^ is the permittivity of free space.

The dielectric loss tangent (tan *δ*) is given by Eq. (4)^36^

4 $$\tan \delta = \varepsilon^{\prime \prime } /\varepsilon^{\prime }$$

The peak of the dielectric loss tangent (tan δ) is associated with the relaxation time (τ) via the relationship^36^.

5 $$\omega \,\tau \, = \,1$$

*ω* is the angular frequency in rad s^−1^ of the applied electric signal.

The formula below was utilized to determine the ac-conductivity^37^:

6 $$\sigma_{{{\text{ac}}}} = \, \varepsilon_{{\text{o}}} \omega \varepsilon^{\prime } \tan \delta$$

Impedance spectroscopy data were used to calculate the contribution of mobility (μ) and charge carrier concentration (n) to the electrolyte’s total conductivity^36,38–40^.

7 $${\text{n}} = \sigma_{{{\text{dc}}}} {\text{k}}_{{\text{B}}} {\text{T}}\tau_{{\text{m}}} \eta /{\text{e}}^{{2}} {\text{L}}^{{2}}$$

8 $$\eta = \varepsilon^{\prime }_{{{\text{LF}}}} /\varepsilon^{\prime }_{{{\text{HF}}}}$$

9 $$\mu = {\text{e L}}^{{2}} /{\text{k}}_{{\text{B}}} {\text{T}}\tau_{{\text{m}}} \eta^{{2}}$$

10 $${\text{D }} = {\text{ k}}_{{\text{B}}} {\text{T}}_{{\mu /{\text{e}}}}$$

k_B_ is the Boltzmann constant, T is the absolute temperature, η is the ratio between the dielectric constant at low frequency (ε′_LF_ ) and high frequency (ε′_HF_), and e is the electric charge.

The complex electric Modulus permittivity (M^*^) of polymeric materials is the inverse of complex relative permittivity and is expressed as the real (M') and imaginary components (M'')^41^, where

11 $${\text{M}}^{\prime } = \omega .{\text{C}}_{{\text{o}}} .{\text{Z}}^{\prime \prime }$$

12 $${\text{M}}^{\prime \prime } = \omega \cdot {\text{C}}_{{\text{o}}} \cdot {\text{Z}}^{\prime }$$

C_o_ is the vacuum capacitance.

Based on Wagner’s polarization technique, the transference numbers for ions (t_ion_) and electrons (t_e_) were determined using the following equations:^42^

13 $${\text{t}}_{{{\text{ion}}}} = \, \left( {{\text{ I}}_{{\text{i}}} {-}{\text{ I}}_{{{\text{ss}}}} } \right){\text{/ I}}_{{\text{i}}}$$

14 $${\text{t}}_{{\text{e}}} = { 1 }{-}{\text{ t}}_{{{\text{ion}}}}$$

I_ss_ represents the steady-state current, while I_i_ denotes the initial current obtained from the plot of current versus time, respectively.

## Results and discussion

### XRD

The X-ray diffraction study is a helpful technique for deciding the structure and crystallization of polymeric materials. Therefore, the XRD patterns of the pristine PVA film and the solid electrolyte films were analyzed and presented in Fig. 1. The diffraction pattern of PVA demonstrates a broad peak at 2θ = 19.98°, which denotes that PVA has a dual amorphous-crystalline class^43^. Integrating PANI with PVA enhanced the amorphous phase because of the interactions between the PANI and PVA polymer chains. The polymer electrolyte samples containing LiI exhibited similar broad, amorphous peaks. Nevertheless, as the concentration of LiI salt increases, the breadth of these peaks also grows due to the disorder introduced in the crystalline phase of PVA. For composite samples with a low LiI content (< 25 wt%), the XRD patterns did not reveal any peaks associated with the LiI salt (2θ = 20.8, 27.7, 32.3°)^44^. This suggests that the salt had fully dissolved within the polymer matrix, expanding the polymer’s amorphous phase. This enhancement in the amorphous structure provides spaces that are easier for ion diffusion and supports the segmental movement of the polymer chains, promoting ion hopping and enhancing ionic conductivity. This is because mobile charge carriers typically have the ability to travel faster in the amorphous area than in the crystalline phase^45–47^. It is crucial to highlight that in the samples with the highest concentrations of LiI (25 wt%), distinct XRD peaks associated with LiI are observed. This suggests that some crystallites of LiI have formed within the PVNLi25 membrane, indicating that not all of the LiI has dissolved in this composite membrane.

### FT-IR

**Fig.2:**
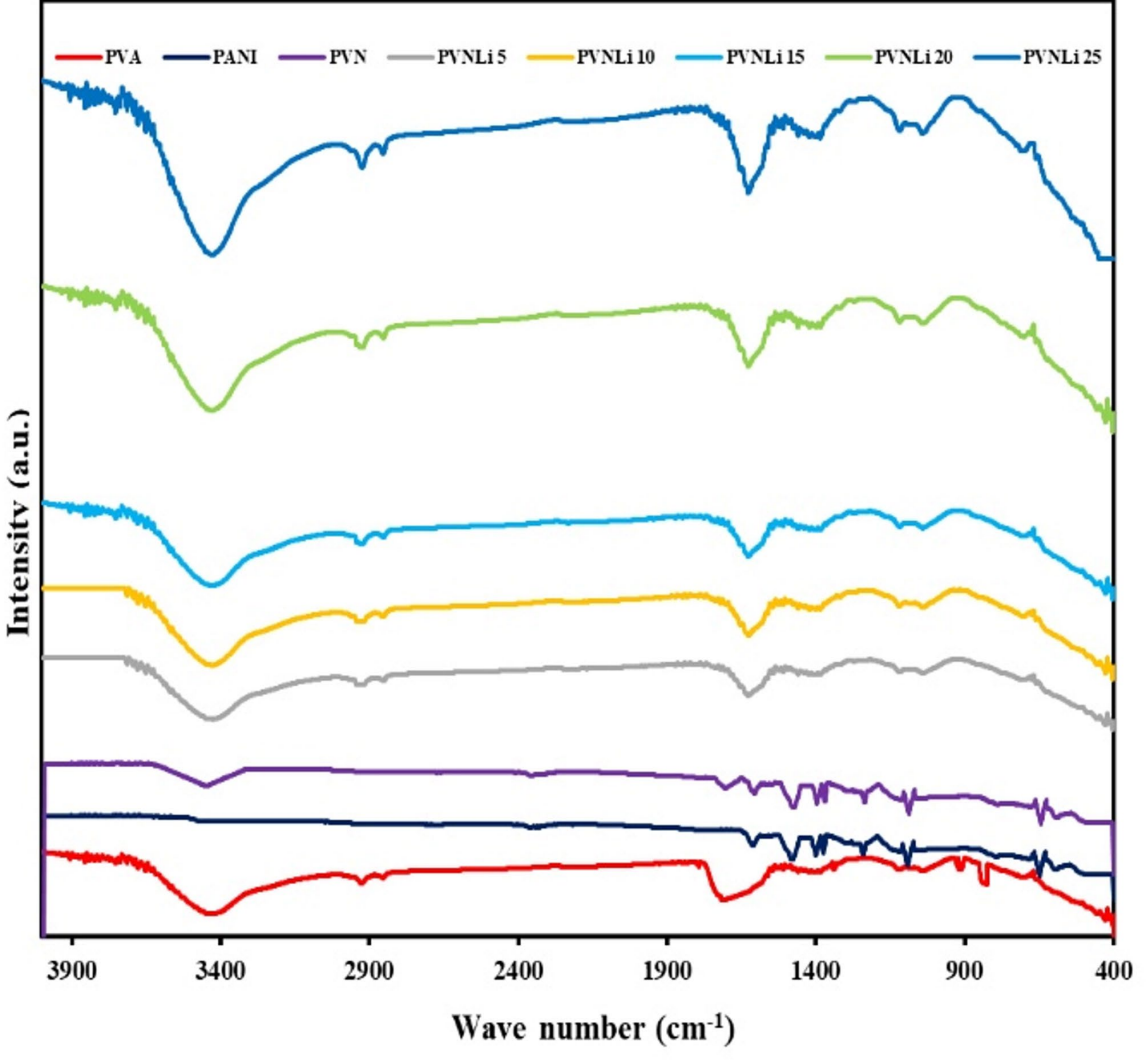
FT-IR of the investigated membranes.

The FTIR spectra of the examined polymer electrolytes have been analyzed to identify the interactions between the PVA chain, PANI, and LiI. The resulting spectra are illustrated in Fig. 2. The PANI peaks related to the C-N stretching were detected at 1239 cm^−1^ and 1375 cm^−1^, while the C = N stretching corresponding to the quinone ring vibration appeared at 1561 cm^−1^^48^. An additional peak was observed at 647 cm^−1^, which matches the out of plane bending vibration of the NH_2_ group in the aromatic amine^49^. In reference to the chemical structure of pure PVA, the absorption peaks at 3412, 2927, 1714, 1420, 1212, 914, and 825 cm^−1^ were noted, corresponding to the stretching of O–H, C–H stretching, C = O stretching from the acetate group of PVA, O–H bending, C-O stretching, C–H wagging, and C–H bending, respectively. The peaks in the 1400 to 1460 cm^−1^ range indicate the vibrations associated with the PVA-PANI composition^50,51^. The decrease in intensity and the shifts in the wavenumber of the O–H and C-O groups in the PVA composite samples, when compared to pure PVA, suggest possible interactions between the salt ions and the proton of PANI, in addition to the polar groups of PVA.

### Mechanical properties

**Fig. 3:**
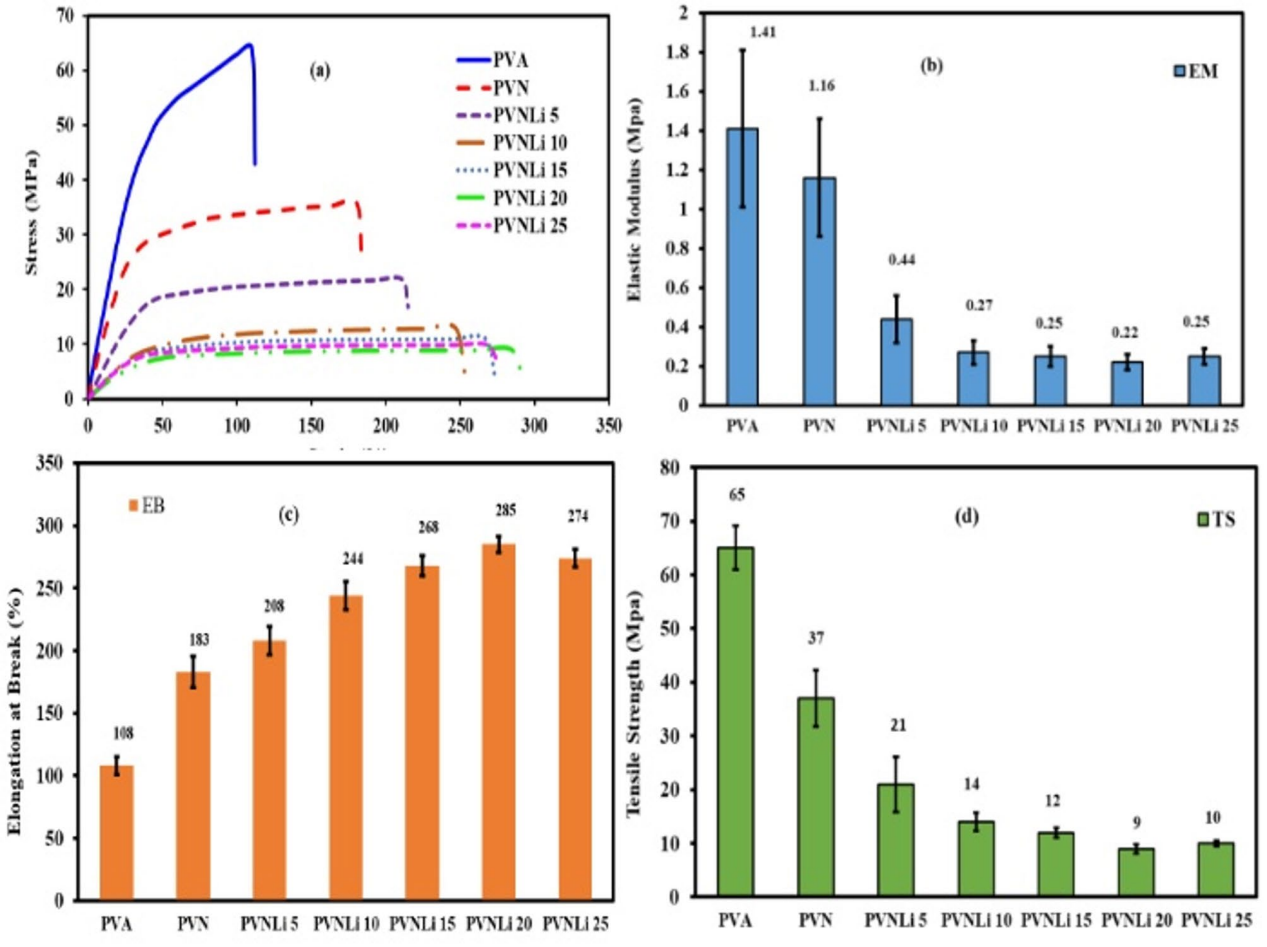
(**a**) Stress–strain graph, (**b**) Elastic Modulus, (**c**) Elongation at Break, (**d**) Tensile Strength for investigated membranes.

The mechanical properties of the tested membrane were examined, and the outcomes are listed in Fig. 3 and Table 2. The table illustrates that virgin PVA exhibits somewhat reduced elongation at Break, a higher elastic modulus, and greater tensile strength than the electrolyte samples. The semicrystalline arrangement and tightly packed polymer chains of PVA are responsible for this^52^. The improvements in the mechanical properties^53^ were amplified by increasing the amount of LiI additives. PANI molecules and LiI insertion into the spaces between PVA chains reduces the number of active centers in the polymer chains, consequently decreasing intra- and intermolecular interactions^54^. This leads to an increase in the free volume, or the gaps between the chains, allowing the polymer chains to move more freely. This increased segmental motion means the chains can stretch and bend more easily, enhancing the composite’s elasticity. Consequently, by adding PANI and LiI, the elongation at the Break of the electrolyte composite samples exceeds that of the undoped PVA film (108 ± 7.1%).

**Table 2:** Mechanical Properties of the investigated membranes.

Sample code	Elastic modulus (MPa)	Tensile strength (MPa)	Elongation at break (%)
PVA	1.41 ± 0.4	65 ± 4.1	108 ± 7.1
PVN	1.16 ± 0.3	37 ± 5.2	183 ± 12.3
PVNLi 5	0.44 ± 0.12	21 ± 5.1	208 ± 11.5
PVNLi 10	0.27 ± 0.06	14 ± 1.7	244 ± 11.4
PVNLi 15	0.25 ± 0.03	12 ± 0.9	268 ± 8.2
PVNLi 20	0.22 ± 0.03	9.0 ± 0.8	285 ± 6.4
PVNLi 25	0.25 ± 0. 03	10 ± 0.5	274 ± 7.2

### Electrical properties

**Fig. 4:**
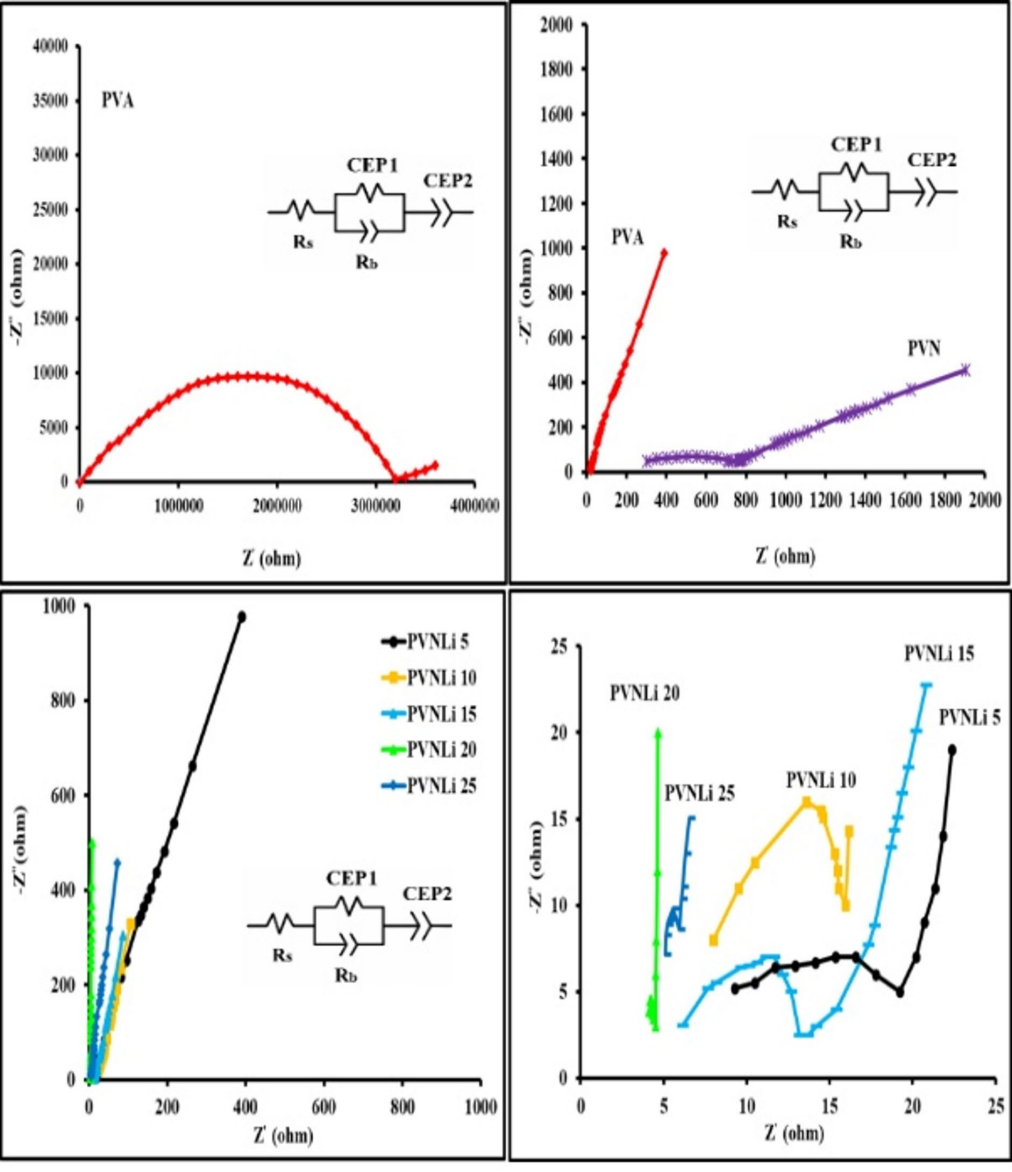
Nyquist plots of the investigated membranes.

EIS is a commonly used experimental method for assessing the ionic conductivity of electrochemical systems. The data generated from the EIS measurements are represented in a Nyquist plot, with the real impedance (Z') displayed on the x-axis, and the imaginary impedance (Z") represented on the y-axis^55^. The Nyquist plot for all electrolyte membranes are illustrated in Fig. 4. The figure indicates that every electrolyte membrane developed exhibits a spike in the low-frequency zone following a depressed semicircle in the high-frequency range. This behavior is linked to the conductivity mechanism that operates across the samples of solid polymeric electrolytes^56^. The semicircular characteristic noted in the high-frequency region is associated with the immobilized polymer matrix, which is typically created by the parallel arrangement of charge transfer resistance (R_ct_) and its related double-layer capacitor (Q). The resistor component is facilitated by the ions’ free mobility within the host matrix, whereas the immobilized ions enable the formation of the capacitor^57^. The detected incline spike in the low-frequency section of Fig. 4 may originate from the polarization effect occurring at the electrode|electrolyte interface. It relates to the Warburg impedance (W), which is associated with ion diffusion’s combined effects across the electrode–electrolyte interfaces^58^. The equivalent circuit representing the EIS plots of the investigated membranes is demonstrated in the inset of Fig. 4.

The bulk resistance of the material is defined as the point where the semicircle ends or the slanted region begins. The semicircle almost vanishes in the highly conductive electrolyte membranes, and only the spike remains. The semicircle’s absence indicates that the polymer’s resistive component is small. An angle of less than 90 degrees between the sloped line and the X-axis signifies that the interface between the electrode and electrolyte is neither uniform nor smooth^59^. The Nyquist plots presented in Fig. 4 illustrate that the diameter of the semicircle in the host matrix (PVN-membrane) reduces as the concentrations of LiI increase, achieving the most significant reduction at 20 weight percent. Beyond that point, it starts to increase. The bulk resistance of the PANI composite membrane decreased because of the increased number of charge carriers, which improves the ionic conductivity of the electrolytes examined. Upon analyzing the impedance data through Z-view software, the equivalent circuit diagram was derived and illustrated in the inset of Fig. 4. This diagram is interpreted as a parallel arrangement of a constant phase element (CPE1) alongside R_b_ for the carriers within the electrolytes, which are connected in series with electrode resistance (R_s_) and another constant phase element, CPE2. The findings are summarized in Table 3. As indicated in Table 3, both CPE1 and CPE2 exhibited an increase with higher LiI concentrations, which corresponds to a greater number density of ions that migrated from the bulk of the electrolyte to the electrodes’ surface. Furthermore, conductivity also rises with increasing LiI concentration. By fitting the impedance data, we calculated the ionic conductivity using Eq. (1), and the findings are presented in Table 4. The table indicates that adding additives (PANI and LiI) to the PVA matrix improves the conductivity value. Since the ionic conductivity depends on the concentration of free ions in the sample, thus it can be said that the observed increase in σ_dc_ within the composite membranes is attributed to the interaction between Li^+^ ions and the OH groups of PVA. The Li^+^ ions act from the charge transfer complex sites as a lattice site for migrating Li^+^ ions in the PVA/PAN composite. Lowering the barrier separating these trapping sites facilitates the movement of Li^+^ ions, creating a pathway within the polymer matrix’s amorphous region, which enhances conductivity^60^. This mechanism occurs due to a rise in the concentration of charge carriers and an enhancement in the amorphous characteristics of the polymer electrolyte, as mentioned earlier in the XRD section. In the analysis of dielectric loss, the mobility enhancement of charge carriers will be covered.

**Table 3:** Equivalent circuit parameters of investigated membranes.

sample	Thickness of membrane samples (μm)	R_s_ (ohm)	R_b_ (ohm)	CPE1 (F)	CPE2 (F)
PVA	40	24	3.2 × 10^6^	2 × 10^–12^	5.1 × 10^–10^
PVN	16	240	4.28 × 10^2^	4.2 × 10^–11^	8.2 × 10^–9^
PVNLi5	2	3.4	11. 7	3.1 × 10^–10^	4.6X10^–7^
PVNLi10	3	3.6	9.2	4.2 × 10^–10^	5.1 × 10^–7^
PVNLi15	6	3.8	7.1	6.7 × 10^–9^	7.2X10^–7^
PVNLi20	16	4.1	0.4	1.1 × 10^–8^	9.4 × 10^–7^
PVNLi25	23	4.5	0.9	5.1 × 10^–9^	7.3 × 10^–7^

**Table 4:** Comparison table of performance polymeric electrolytes.

Polymer	Salt	Conductivity σ (S cm^−1^)	Reference
PVA	Mg(NO_3_)	7.36 × 10^–7^	^61^
PANI	LiClO_4_	2.44 × 10^–4^	^62^
PVA-PVdF	LiClO_4_	3.03 × 10^–5^	^63^
PVdF-PVA	LiTFSI	4.31 × 10^–4^	^64^
PANI	LiPF	3.7 × 10–3	^65^
PANI	LiCF_3_SO_3_	1 × 10^–3^	^66^
PANI-PVA	LiClO_4_	2.5 × 10^–4^	^67^
PVA-PVdF	LiCF_3_SO_3_	2.7 × 10^–3^	^68^
PVA	NH_4_Al(SO_4_).12H_2_O	1.73 × 10^–5^	^69^
PVA	Cu(NO_3_)_2_	1.6 × 10^–5^	^70^
PVdF-PANI	NaHCO_3_	3.32 × 10^–3^	^71^
PVA-PANI (PVNLi20)	LiI	4.46 × 10^–3^	Present work

It is important to note that numerous polymers have been studied in the literature for developing polymeric electrolytes with various electrolyte salts, such as polyacrylonitrile (PAN), polypropylene oxide (PPE), polymethyl methacrylate (PMMA), polyethylene oxide (PEO), polyvinyl chloride (PVC), polyvinylene difluoride (PVdF), and polyaniline (PANI). Table 4 presents a comparison of the performance of these polymeric electrolytes against PVNL20, which is our top-performing polymeric electrolyte sample. It is clear that our cost-effective sample exhibits a higher conductivity value than most other polymeric electrolyte samples.

### Dielectric spectra analysis

Dielectric analysis serves as an effective method for examining the conduction mechanisms within polymer composites and the interactions between ions and polymers. The theory of polarization suggests that applying an electric field leads to the electric polarization of dielectric materials. The dielectric constant (ε') and dielectric loss (ε″) for the membranes tested were derived based on Eqs. (3, 4).

**Fig. 5:**
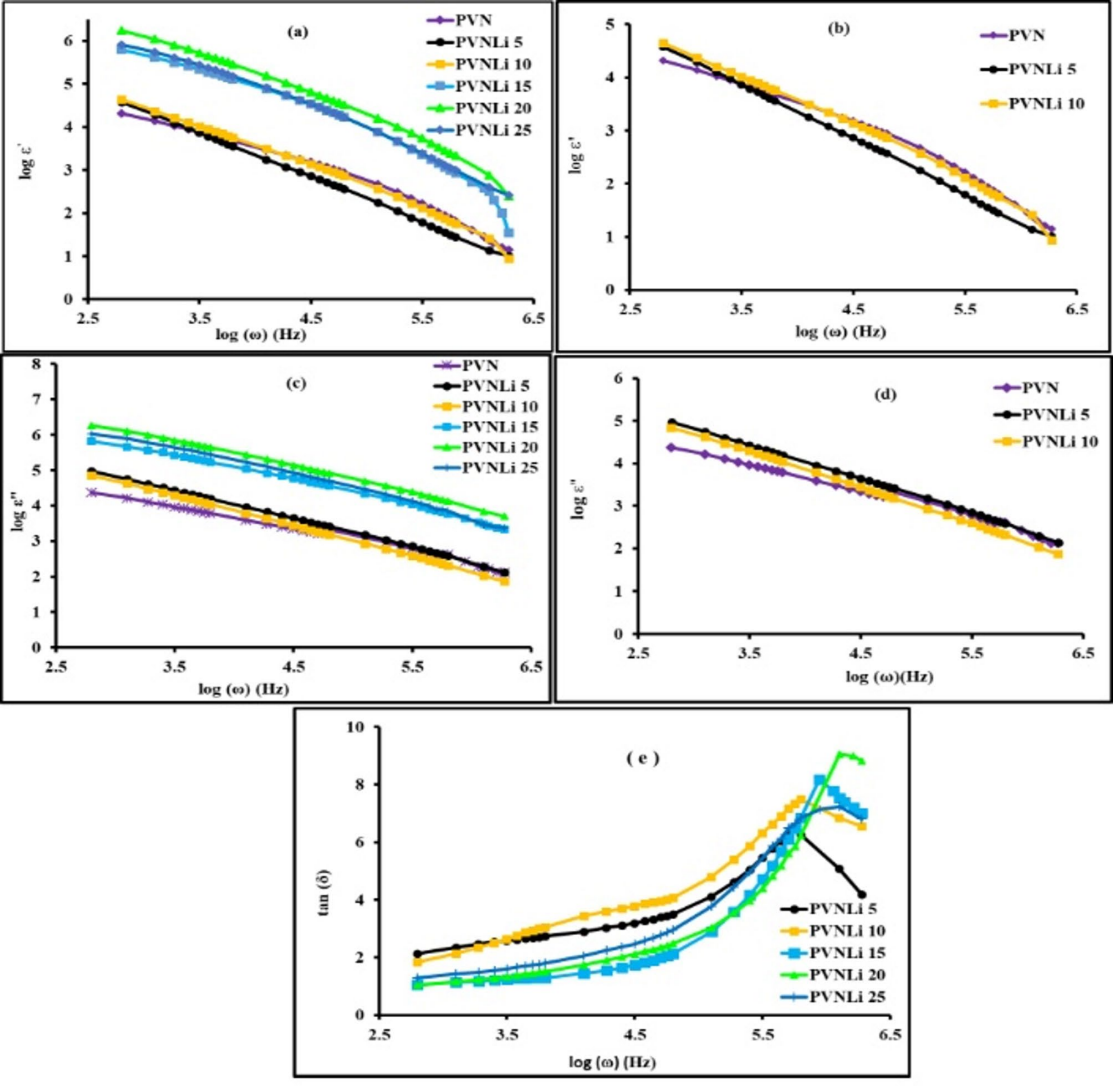
Frequency dependence of dielectric constant (**a**,**b**), dielectric loss (**c**,**d**) and tangent loss (**e**) of investigated membranes.

Figure 5 (a-d) demonstrate the variation of the dielectric constant and dielectric loss of pure PVA and LiI-doped PVA/PANI composites as a function of frequency at room temperature. Adding LiI led to a higher dielectric constant in the studied films, reaching a maximum of 20 wt%. This phenomenon underscores the effect of electrode polarization and Maxwell-Wegner ionic conduction within the PVA composite, which is related to the existence of –OH polar groups. This behavior can be explained by understanding that extra charge carriers are unable to move through the blend due to the movement of lithium ions. Figure 5 (a,b) illustrates that all samples exhibit high ε'- values in the low-frequency range, which supports the non-Debye type behavior caused by charge accumulation at the electrode|electrolyte interface. The ε'- value decreased quickly with increasing frequency because the charge carriers could not adjust to the field direction in a timely manner. However, at higher frequencies, the dielectric constant showed little dependence on frequency since the charge carriers cannot realign with the field direction. Based on the unique characteristics of dipolar relaxation, the dielectric loss factor can be used to measure the frequency and strength of relaxation. Figure 5 (c,d) illustrates the dielectric loss (ε'') spectra for pure PVA films and their composites. The figures indicate that dielectric loss reduces with an increase in frequency. By incorporating LiI in amounts up to 20 wt%, the ε''-values in the low-frequency range rose. The higher ε''-values imply an enhancement in the movement of the material’s free charge carriers.

The analysis of the dielectric tangent Tan (δ) was used to study the relaxation processes of the tested materials^72–74^. The room temperature tan (δ) values at different frequencies were determined using Eq. (4): tan (δ) = ε''/ ε'. The outcomes are shown in Fig. 5(e). The identification of the peak in the tan δ value signifies the presence of dielectric relaxation**.** This peak moved toward higher frequencies as the Li concentration rose to 20 wt%. The peaks depicted in Fig. 5(e) correspond to the ionic translational dynamics linked to the relaxation of mobile ion conductivity. This observation reinforces the notion of ion transport facilitated by the segmental mobility of the electrolytes^75,76^. Given that active (ohmic) elements are more dominant than reactive (capacitive) elements, it is observed that tan δ increased with frequency. Subsequently, the reduction in tan δ at higher frequencies is likely attributable to the active element’s diminishing influence and the reactive element’s increasing influence^77^. The tan δ plot suggests a non-Debye behavior of the system, which characterizes the electrolyte relaxation phenomenon^75^. The (tan δ)_max_ value was utilized to ascertain the angular frequency of the relaxation peak (ω_peak_). This data was then employed to calculate the relaxation time (τ) for each electrolyte using Eq. (5). The computed values are displayed in Table 5.

**Table 5:** Electrical data of the investigated membranes.

Sample	σ_dc_ (Ohm^−1^ cm^−1^)	R_b_ (Ω)	n (cm^−3^)	μ(cm^2^s^−^V^−1^)	D (cm^2^s^−1^)	τ ×10^6^ (s)	τ^+^	Decomposition Potential (V)
PVA	1.5 × 10^–9^ ± 1.1 × 10^–11^	3,200,000 ± 41000	–	–	–	–	–	–
PVN	4.47X10^–7^ ± 4 × 10^–10^	427.59 ± 6.12	–	–	–	–	0.774	–
PVNLi 5	4.67 × 10^–6^ ± 3.8 × 10^–8^	11.71 ± 0.25	1.45 × 10^18^	2.44 × 10^–5^ ± 1.2 × 10^–7^	6.32X10^–7^ ± 6.2 × 10^–9^	1.77 ± 0.03	0.941	1.98 ± 0.02
PVNLi 10	8.91 × 10^–6^ ± 9 × 10^–8^	9.16 ± 0.18	2.76 × 10^18^	4.41 × 10^–5^				
± 4.3X10^–7^	1.13 × 10^–6^							
± 1.2X10^–8^	1.58 ± 0.04	0.974	2.17 ± 0.02					
PVNLi 15	9.33 × 10^–5^ ± 1 × 10^–6^	7.05 ± 0.15	4.51 × 10^18^	6.07X10^–5^ ± 7 × 10^–7^	3.56 × 10^–6^ ± 3.6 × 10^–8^	1.14 ± 0.04	0.978	2.17 ± 0.03
PVNLi 20	4.46 × 10^–3^ ± 5 × 10^–5^	0.42 ± 0.08	1.61 × 10^19^	2.36 × 10^–3^ 2.4 × 10^–5^	6.13 × 10^–5^ ± 6.2 × 10^–7^	0.71 ± 0.03	0.997	2.21 ± 0.02
PVNLi 25	3.16X10^–3^ ± 4 × 10^–5^	0.87 ± 0.03	1.04 × 10^19^	1.91 × 10^–3^ 2.1 × 10^–5^	4.91 × 10^–5^ ± 5.5 × 10^–7^	1.09 ± 0.04	0.997	2.18 ± 0.03

According to the results discussed earlier, the calculations for number density (n), mobility (μ) of free ions, and the diffusion coefficient of the ions were carried out using Eqs. (7–10), with the resulting parameters shown in Table 5.

### AC-conductivity

**Fig. 6:**
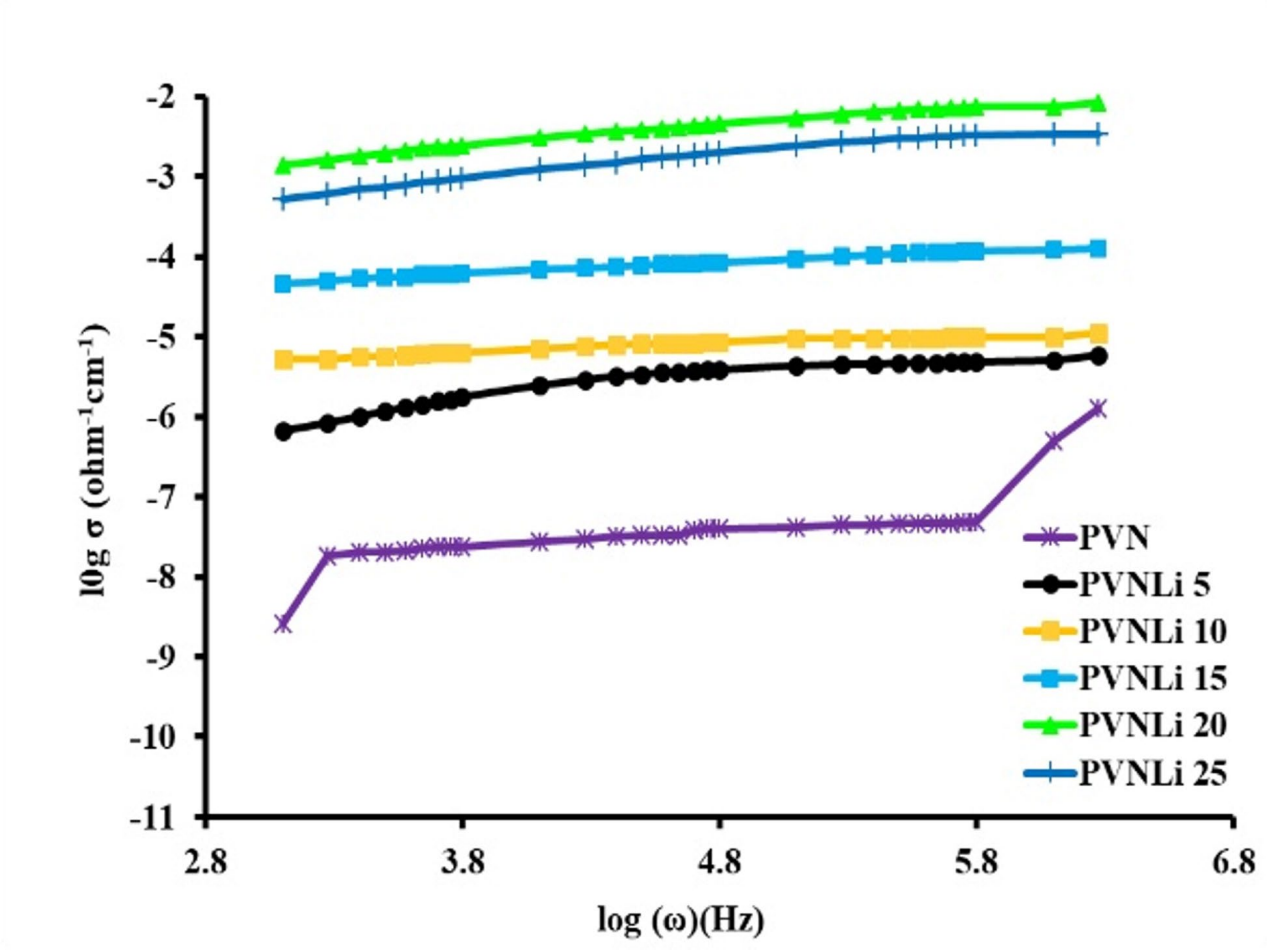
Frequency dependence plots of ac-conductivity of the investigated polyelectrolyte membranes.

The AC conductivity (σ_ac_) of the tested membranes was calculated from the impedance data measured at room temperature and over a frequency range of 10^2^ – 3 × 10^5^ Hz using Eq. (6). The outcomes are illustrated in Fig. 6. This figure features three separate sections: (I) The low-frequency section, which illustrates the polarization effect taking place at the electrode/electrolyte interface. In this part, mobile charge carriers were accumulated at the interface, reducing ionic conductivity. (II) The mid-frequency section reveals a constant conductivity regardless of frequency; therefore, the conductivity values measured in this segment are considered to be DC conductivity. (III) The high-frequency section depicts increased conductivity, which is associated with short-range ion movement. In this area, conductivity rose due to a boost in displacement current as capacitive reactance declined. Lithium ions’ simultaneous forward and backward movements in this region result in a rise in ionic conductivity as the frequency increases. In pure polymers and composites with low salt concentrations, the three zones are clearly visible, as is typical for polymer electrolytes^78,79^; however, the high-frequency dispersion area disappears when additives are added to the PVA matrix^77^. The low-frequency dispersion zone migrated to higher frequencies and became more noticeable as the additive concentration rose. This is explained by the growth of free charge carriers that are available inside the membrane. This is a result of improvements made to the system’s free-charge carriers. The DC conductivity values were obtained by extrapolating the plateau regions of the analyzed membranes down to zero frequency. The acquired results are listed in Table 5.

### Electric modulus study

**Fig. 7:**
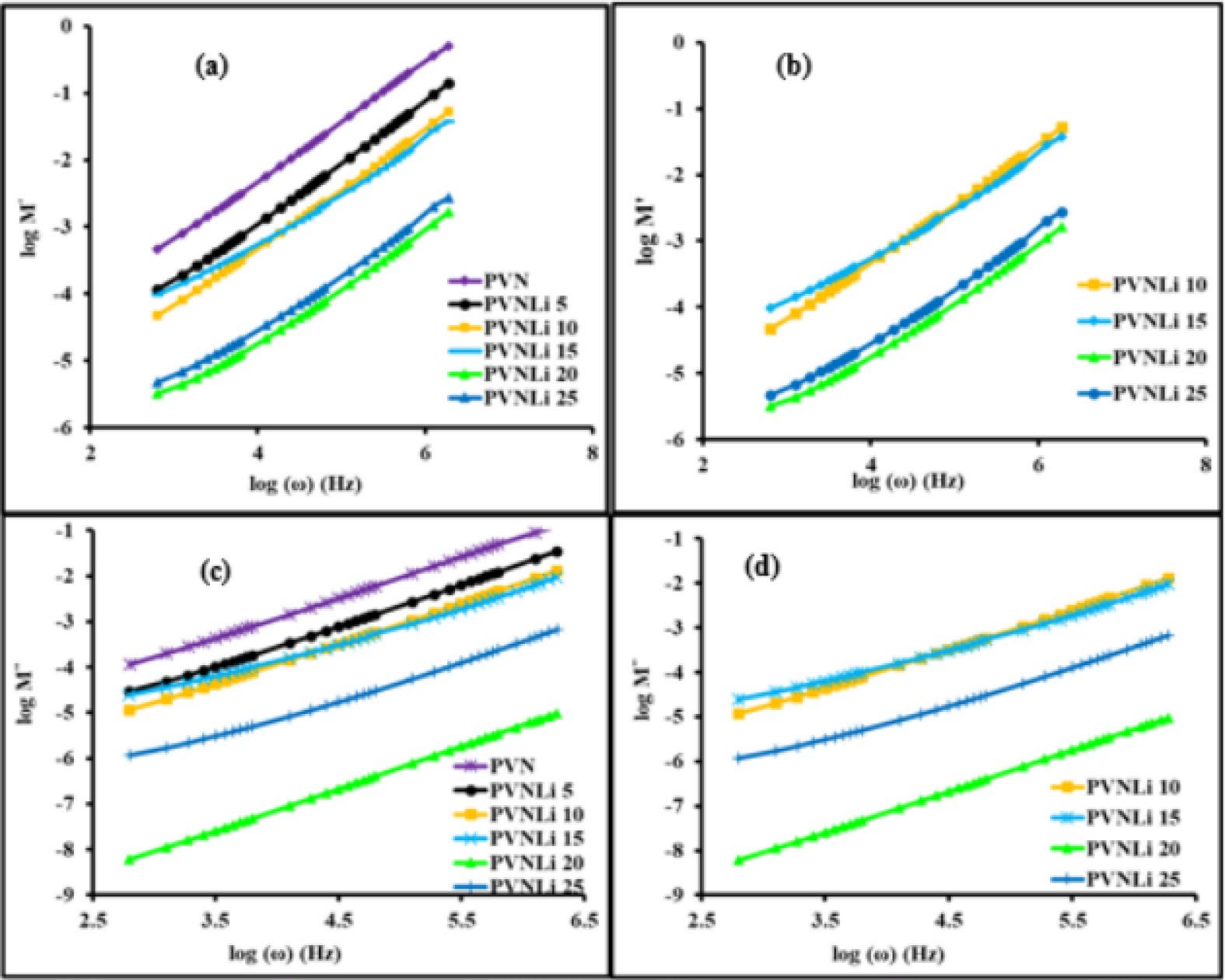
Frequency dependence of electric modulus, M/ (**a**,**b**) and imaginary electric modulus, M// (**c**,**d**), at room temperature for investigated electrolyte membranes.

Analyzing the electrical modulus is beneficial for distinguishing between electrode polarization and different interfacial phenomena^80^. Therefore, the electrical moduli (M' and M’') were evaluated at room temperature across a range of log frequencies for various concentrations of LiI, with the outcomes depicted in Fig. 7 (a and b). Both graphs show that M’ and M’' rise as the frequency increases. The M’ value at lower frequencies tends toward zero, indicating that electrode polarization has a minimal effect^81^. The elongated tail observed at lower frequencies is associated with the high capacitance related to the electrodes and indicates that the behavior deviates from the Debye model^82^. The dispersion of M’ at higher frequencies results from relaxation conductivity, and the restricted range of charge carriers’ mobility can explain the continual increase in M’ values with increasing frequency. In electrolyte membranes, the migration of ions between sites disturbs the electric potential in their surroundings, influencing adjacent ions’ movement. The collective movement of ions results in a decay or conduction process that does not follow an exponential pattern defined by various relaxation times^83^. The maximum value of M′ observed for 20 wt% LiI can be attributed to its lower dielectric constant than other samples.

All the membranes tested indicated that M″ has small values at lower frequencies, probably due to the significant capacitance arising from the electrode polarization effect. This phenomenon results from accumulating numerous charge carriers at the boundary between the electrode and the polymer.

### Transference number analysis (TNM)

**Fig. 8:**
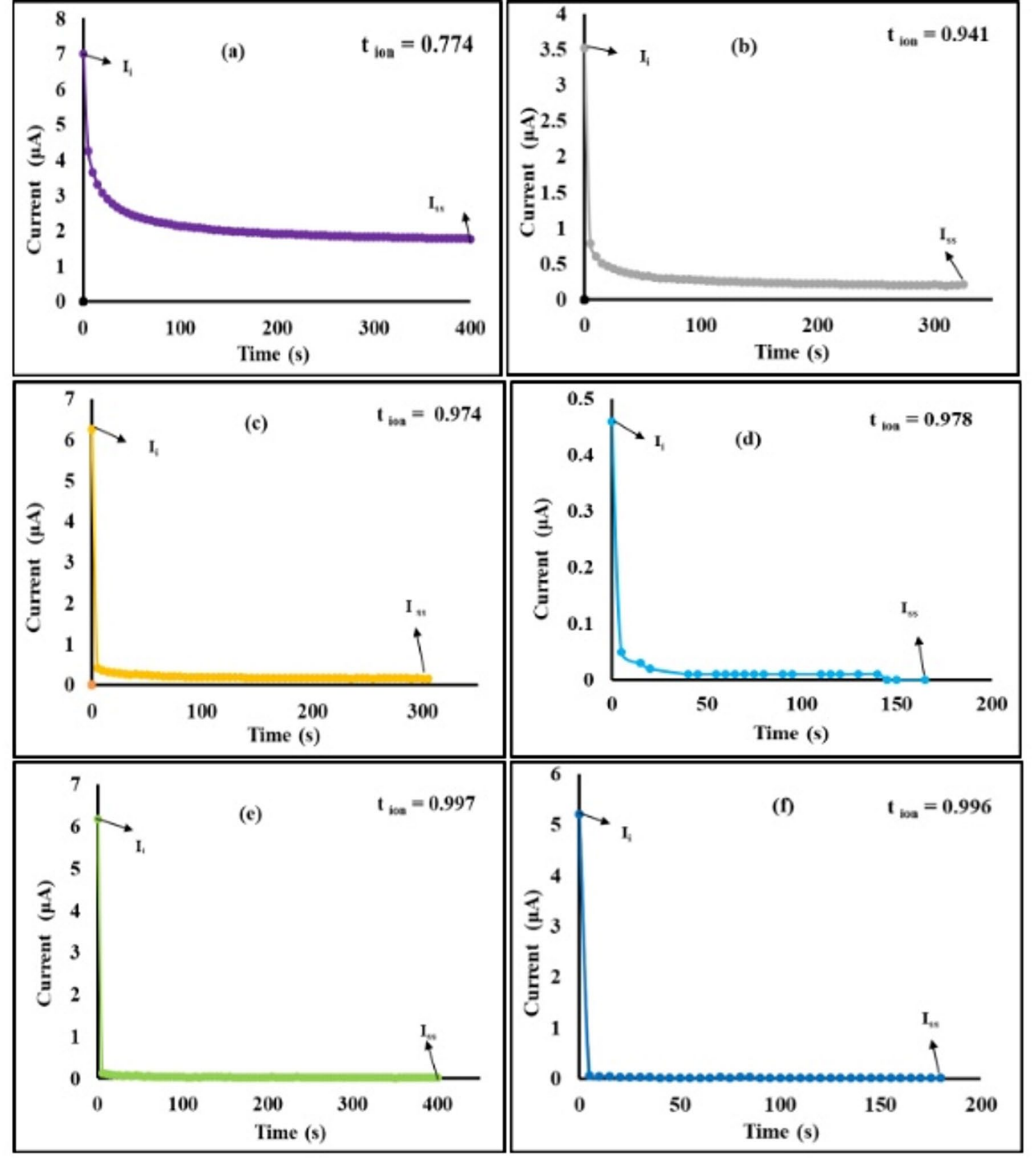
Electric current as function of time under polarized voltage of 0.2V (**a**) PVN, (**b**) PVNLi 5, (**c**) PVNLi 10, (**d**) PVNLi 15,(**e**) PVNLi 20 , (**f**) PVNLi 25.

The contribution to ionic conductivity of the evaluated electrolyte membranes was analyzed by applying a constant DC voltage of 0.2 V in a setup featuring a blocking electrode cell consisting of SS| electrolyte| SS. The changes in polarization current over time were monitored and are depicted in Fig. 8. The figure shows that the initial current (I_i_), which reflects the contributions from both ions and electrons, decreases rapidly over time and stabilizes at a constant value (I_ss_) once the electrolyte has been fully depleted. The ionic (t^+^) and electronic (t^−^) transference numbers in the membranes were calculated using Wagner’s DC polarization method, utilizing Eqs. (13) and (14). The outcomes are presented in Table 5, indicating that the ionic transport number (t^+^) ranks as follows: PVNLi20 > PVNLi25 > PVNLi15 > PVNLi10 > PVNLi5 > PVN.

### Electrochemical stability

**Fig. 9:**
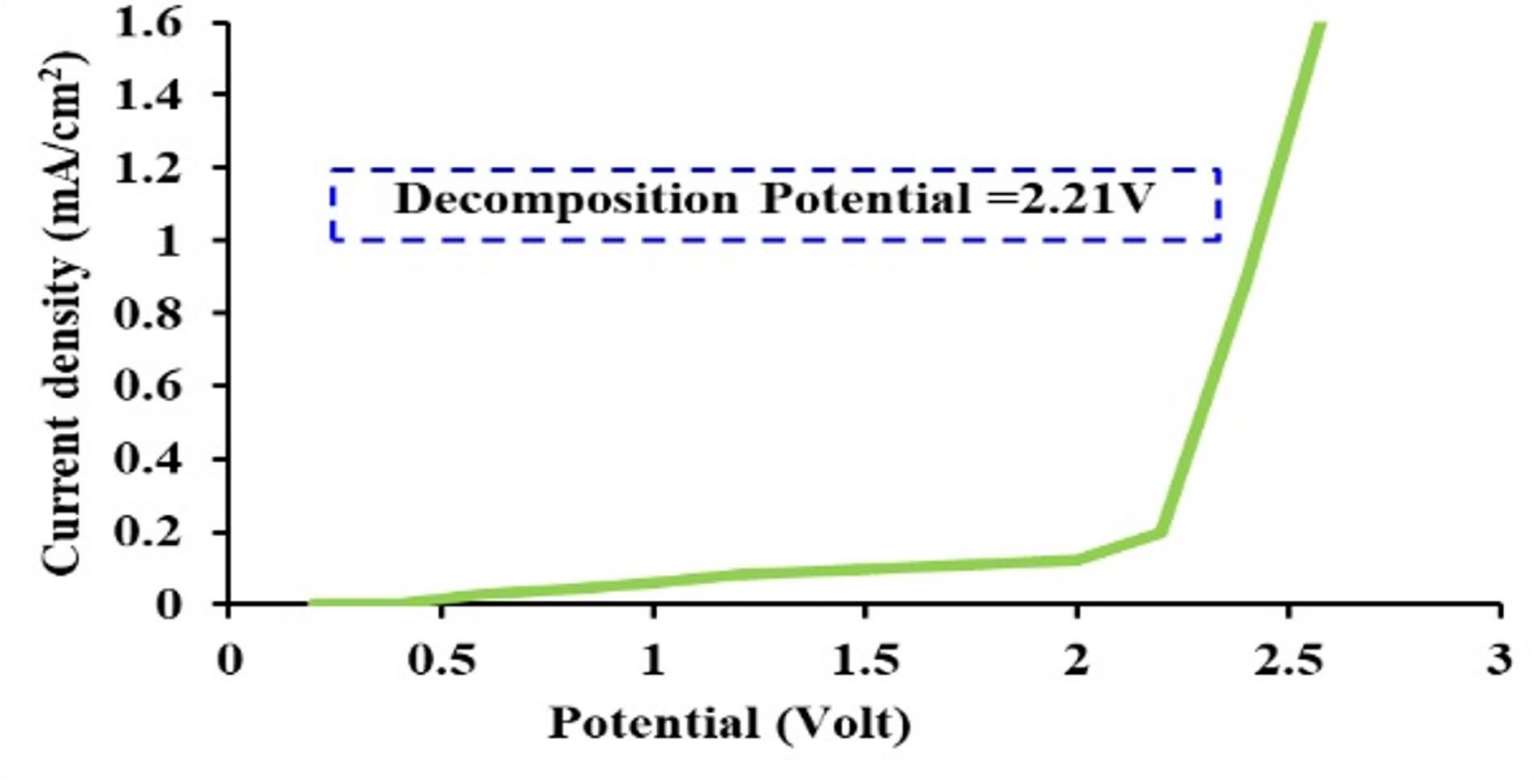
LSV curve for the highest conducting polymer electrolyte (PVNLi 20).

The LSV method is a simple way to identify the potential widow of a polymer electrolyte prior to its application in energy storage devices. An SS| electrolyte| SS cell was utilized to determine the breakdown voltage. Figure 9 illustrates the LSV response of the PVNLi 20 solid electrolytes, which exhibit relatively high conductivity. The LSV curve indicates that no significant current density was detected up to 2.21 V, suggesting that the electrochemical reaction did not take place within the electrolyte. Once the voltage exceeds 2.21 V, the polymer electrolyte’s breakdown voltage (V_d_) is reached. The results of other membranes are listed in Table 5, which indicates that the relatively high-conducting electrolyte investigated in this study has the potential stability to be employed in energy storage applications.

## Conclusions

The research focused on developing PVA, PVA-PANI blends, and PVA-PANI-LiI polymer electrolytes through solution casting techniques. XRD analysis revealed that the incorporation of LiI and PANI into PVA yields an amorphous structure, with crystalline peaks identified in the PVNLi25 membrane (containing 25% LiI), indicating the presence of LiI crystals. Moreover, the mechanical properties showed improvement with increasing LiI content, as the elongation at break for the composite samples exceeded that of the undoped PVA film (108 ± 7.1%). Higher LiI concentrations in the polymer electrolytes enhanced conductivity and reduced bulk resistance, reaching a maximum at 20 wt%. The PVNLi20 membrane exhibited the highest conductivity of 4.1 × 10⁻^3^ S cm⁻^1^ along with good electrochemical stability (2.21 V) and an ionic transference number of 0.997. The study indicates that electrode polarization raises the dielectric constant of all electrolyte membranes at lower frequencies. The frequency-dependent electric modulus and dielectric loss reveal significant interactions between segmental and ionic movements within the polymer. The loss tangent indicates broad peak distributions, pointing to deviations from Debye relaxation. These results suggest that PVA-PAN-LiI nanocomposite membranes have the potential to function as efficient electrolytes in supercapacitors and various energy storage systems.

## Supplementary Information

Below is the link to the electronic supplementary material.

Supplementary Material 1

## Data Availability

The datasets used and/or analyzed during the current study are available from the corresponding author upon reasonable request.
