# Supplementary material for: Electrical properties of PVA/PANI/LiI composite membranes
Source: Sci Rep. 2025 Dec 18;15:44131. doi: 10.1038/s41598-025-30722-x (PMC12717162; doi:10.1038/s41598-025-30722-x)
Supplement: Supplementary file 1 — Supplementary Material 1 [file 41598_2025_30722_MOESM1_ESM.docx]

**Electrical Properties of PVA/PANI/LiI Composite Membranes**

**A. A. Eissa^1^,** [**E. M. Kamar**](https://link.springer.com/search?facet-creator=%22E.+M.+Kamar%22)**^1^, M. A. Mousa^1^, M. Sameeh^1^**

^1^Chemistry Department, Faculty of Science, Benha University, Benha, Egypt, 13511.


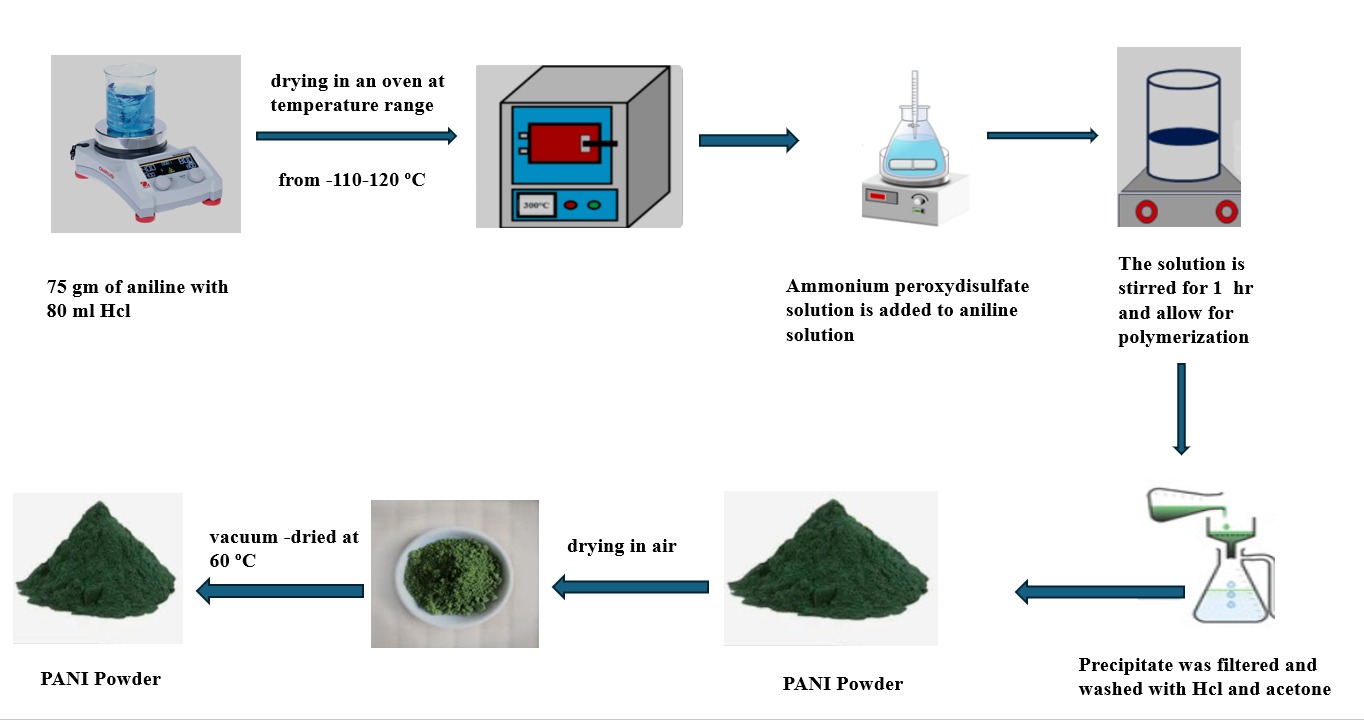


**Fig.S1.** Synthesis of polyaniline (PANI) via oxidative polymerization of aniline hydrochloride with ammonium peroxydisulfate (APS)**.**
